# Risk factors in adolescence as predictors of trajectories of somatic symptoms over 27 years

**DOI:** 10.1093/eurpub/ckac081

**Published:** 2022-07-29

**Authors:** Noora Berg, Tapio Nummi, Christopher G Bean, Hugo Westerlund, Pekka Virtanen, Anne Hammarström

**Affiliations:** Department of Public Health and Welfare, Finnish Institute for Health and Welfare, Helsinki, Finland; Department of Public Health and Caring Sciences, Uppsala University, Uppsala, Sweden; Faculty of Information Technology and Communication Sciences/Statistics, Tampere University, Tampere, Finland; Department of Public Health and Caring Sciences, Uppsala University, Uppsala, Sweden; School of Psychology, The University of Adelaide, Adelaide, SA, Australia; Department of Psychology, Stockholm University, Stockholm, Sweden; Faculty of Social Sciences, Tampere University, Tampere, Finland; Institute of Environmental Medicine, Unit of Occupational Medicine, Karolinska Institutet Stockholm, Sweden; Department of Epidemiology and Global Health, Umeå University, Umeå, Sweden

## Abstract

**Background:**

Somatic symptoms among adolescents are common, yet little is known about long-term trajectories of somatic symptoms and the factors in adolescence that shape them. We examined individual, family and school-based factors at age 16 as predictors of trajectories of somatic symptoms over 27 years.

**Methods:**

Participants from the Northern Swedish Cohort (*n* = 1001) responded to questions about individual factors (e.g. health behaviours), family factors (e.g. contact with parents, social and material adversity) and school satisfaction at age 16; as well as 10 somatic symptoms at ages 16, 18, 21, 30 and 43. Teacher assessments at age 16 included overall ability at school and peer relations. Age 16 predictors of somatic symptom trajectory group membership were analysed using multinomial logistic regression.

**Results:**

Poor contact with mother and poor school satisfaction were significant predictors of adverse symptom trajectories among both men and women. Low birth weight and low parental academic involvement were contributing factors for women, while smoking and social adversity were more relevant factors for men.

**Conclusions:**

Our findings emphasize the importance of a holistic approach that considers the unique contributions of individual, family and school-based factors in the development of trajectories of somatic symptoms from adolescence to middle age.

## Introduction

The prevalence of somatic symptoms, such as headaches, nausea and stomach aches, increases from childhood to adolescence.^1^ Somatic symptoms are often referred to as ‘functional’ when there is no medical explanation for them.^1^^,^^2^ Depending on their number, frequency and severity, somatic symptoms may have adverse psychological and social consequences, such as absence from school, reduced academic achievement^3^ and impaired work ability.^4^ Besides individual suffering and challenges in differential diagnostics, somatic symptoms bring a substantial burden to health care.^5^^,^^6^ Thus, deeper understanding of the development of symptoms will contribute to improvement in health services. Somatic complaints may also predict problems in mental health later in life.^1^^,^^7^^,^^8^ Women usually report more symptoms than men.^9^

Adolescence and young adulthood are the years when the foundations for health are laid, and those foundations tend to determine health trajectories across the life course.^10^ Although the high prevalence of somatic symptoms in adolescence has been suggested to reflect adolescence as a challenging developmental phase of life,^1^ prolonged symptoms, in turn, are health problems in themselves, but may also be an indicator of other (undiagnosed) long-term health problems. Although psychosocial factors, such as the adolescent’s relationship with parents and peers^11^ are evidently associated with somatic symptoms, few studies have examined prospectively predictors of somatic symptoms over time or assessed how the course of somatic symptoms develops from adolescence onwards. Moreover, also a contextual approach is important^12^ (covering both individual and contextual aspects), especially when studying adolescents for whom everyday life settings such as family and school are of vital importance.^13^ The socioecological model may be used to understand the impact of various ecological levels on the developing individual.^12^ On the microlevel, referring to an individual’s immediate environment, two important settings are family and school. The mesolevel refers to interactions between microlevel settings. Macrolevel society is also important to acknowledge, but not the focus of this study. Factors in adolescents’ immediate environment such as peer relational victimization^14^ and a change in family structure (e.g. parental divorce)^15^ have been found to predict later somatic symptoms, although with relatively short follow-up times. These studies have either adjusted for gender or not found any gender differences. Some studies have focused on, for example either somatic or mental health, substance use or social factors, but a holistic approach covering several contextual dimensions of life such as health (weight), health behaviour (smoking, alcohol use), social relations (parental and peer relations, school environment) and socioeconomic factors (material adversity) etc. has been lacking.

When examining the predictors of somatic symptoms, it is important to acknowledge the heterogeneity in the age curve of somatic symptoms and follow individuals across multiple waves to detect individual trajectories of symptoms. To our knowledge, only five studies have specifically estimated trajectory groups of somatic symptoms at several assessment points over time. Three of these studies were limited to childhood and adolescence (follow-up times 2–5 years). Two of them identified four groups with low, increasing, decreasing and high trajectories,^16^^,^^17^ and one identified three groups: one high and two decreasing groups.^18^ Membership to a long-term high symptoms trajectory group was associated with the children experiencing more stress and negative life events, parenting stress at baseline and the child’s previous health, such as depressive symptoms, but not overprotective parenting style compared to low or decreasing groups.^16–18^ Our own two previous studies on somatic symptoms^19^^,^^20^ have covered the life course up to middle age. These studies, however, concentrated on methodology (multiple response trajectory analysis), and did not investigate predictors or outcomes. In our previous study, we identified four trajectories of somatic symptoms from the age 16–43 (‘constantly low’, ‘increasing’, ‘decreasing’ and ‘constantly high’ symptom load).^20^ In accordance with previous studies, the prevalence of symptoms was higher for women in our studies compared to men, but the development of symptoms was similar among both genders.^20^ It is unclear whether there are gender differences in the predictors of symptom trajectories.

In the present study, we address the limitations of previous research by extending the follow-up time window of somatic symptoms from adolescence to nearly three decades. Furthermore, in accordance with ecological theories, health and well-being over the life course are determined not only by individual characteristics but also the broader context such as immediate family environment and school environment.^12^ As such, we examined the association of individual (including gender), family and school characteristics among 16-year-old adolescents with their 27-year trajectories of somatic symptoms. Specific research questions were: (i) ‘Are individual, family and school characteristics associated with trajectory groups of somatic symptoms from age 16–43 among men and women?’ and (ii) ‘Which of the characteristics are the most important predictors of symptom trajectories among men and women?’.

## Methods

### Participants and procedures

The principal investigator approached all pupils (*N* = 1083) attending (or who should have attended) their final year of compulsory school (grade 9, age 16) in 1981, from all schools in Luleå, a middle-sized industrial town in Northern Sweden (the Northern Swedish Cohort).^21^ In total, 506 girls and 577 boys were invited to participate, and 1080 (99.7%) of them participated in the baseline investigation. Follow-up surveys were conducted in 1983 (age 18, *N* = 1077, 99.7% of the baseline participants), 1986 (age 21, *N* = 1060, 98.2%), 1995 (age 30, *N* = 1046, 96.9%) and 2008 (age 43, *N* = 1010, 93.5%). At each phase, the respondents completed an extensive questionnaire on working and school life (during adolescence), family life, health and well-being. In 1981, there was also an interview with the students’ teacher (*N* = 46, none refused) about each pupil’s school performance and adjustment to school. The cohort is comparable to Sweden as a whole with regard to socio-demographic and socio-economic factors as well as health status and health behaviour.^22^^,^^23^ All participants were informed of the study purpose and participation was voluntary. They were requested to indicate their consent by answering the survey questionnaire. Ethical approval was provided by the Swedish Ethical Review Authority. The present data comprised 1001 participants (519 boys, 482 girls) with data available for trajectory analysis.^20^

### Measures

‘Individual factors’ included birth weight (from health care records), further categorized as <2500 g vs. ≥2500 g; from the survey at age 16, current smoking or snuff use (‘yes’/‘no’), having ever been drunk (‘yes’/‘no’) and early experience of being drunk at age <14 (‘yes’/‘no’). Weight and height at age 16 were measured by the school nurses and body mass index (kg/m^2^) was calculated and further dichotomized as ‘overweight’ (≥25 kg/m^2^) vs. ‘not’.

‘Family factors’ were based on the survey at age 16 and included contact with mother and contact with father, based on questions ‘How is your contact with mother/father?’ with 5 response alternatives from ‘very good’ to ‘very poor/no contact’. These were further dichotomized as good vs. average/poor. Respondents with a deceased mother or father were excluded from these analyses, respectively. Parental academic involvement was based on teachers’ ratings of parental interest in the child’s studies (a 5-point scale from ‘very large’ to ‘very small’), and student’s self-report about parental assistance with homework, (a 5-point scale from ‘yes, always’ to ‘no, never’). The variables were dichotomized into good vs. average/poor, and parental academic involvement was indicated if either of the two questions was rated as ‘good’. Social and material adversity were composite scores based on survey at age 16, constructed as previously reported.^24^ Social adversity was indicated if the respondent had experienced at least one of the following: parental loss, residential instability (moving residence more than two times during lifetime), or parental illness (mother’s or father’s somatic illness, mental or alcohol use disorder). Material adversity was indicated if the respondent had at least one of the following: unemployment of mother or father during the previous 12 months, poor material standard of living (less than 3 material items in the family’s possession, from a list of ten items, e.g. colour television and car) and lack of an own bedroom.

‘School-based factors’ included self-rated dissatisfaction with the time spent at school, the lessons and the classmates (5-point scales from ‘very much’ to ‘very little’). A mean score was calculated from these factors and the highest quartile indicated school dissatisfaction. Teachers’ evaluation of pupil’s overall performance or talent (5-point scale ranging from ‘very good’ to ‘very poor’) was dichotomized as good/average and poor. Teacher-rated peer problems included 6-point scales for tendency to isolation vs. extraversion, and non-popularity vs. popularity. A mean score of those two characteristics was calculated and dichotomized by the lowest quartile to indicate the presence of peer problems.

**Figure 1:**
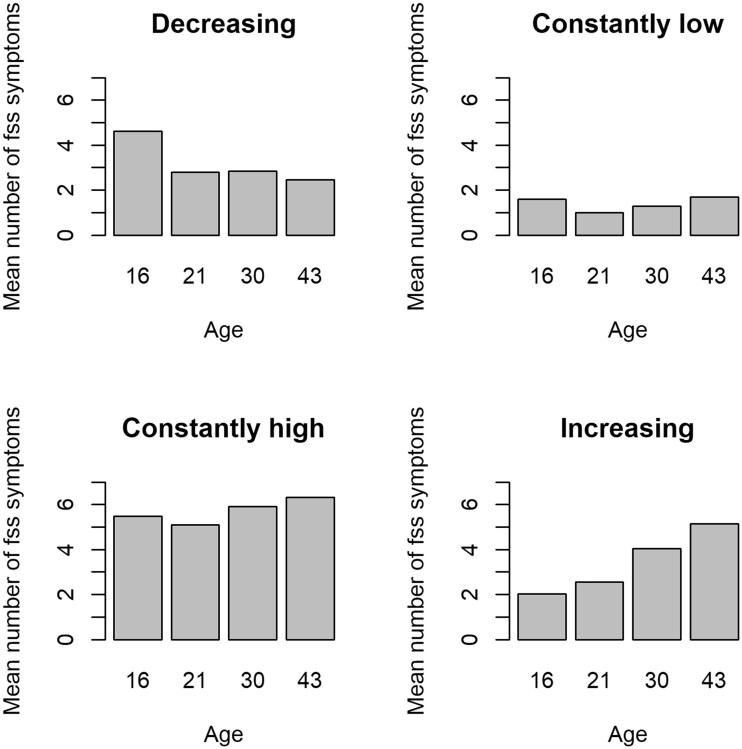
Mean number of somatic symptoms by age for trajectory groups

‘Membership of somatic symptom trajectory group’ was the outcome of this study. The trajectory analysis has been described in detail previously.^20^ Briefly, the presence of the following 10 somatic symptoms during the past 12 months (rated as ‘no’; ‘yes, light’; and ‘yes, severe’) were used to construct a score at each survey (ages 16, 18, 21, 30 and 43): stomach ache other than heartburn, gastritis or gastric ulcer; headache or migraine; fatigue; dizziness; palpitations; nausea; sleeplessness; backache, hip pain or sciatica; breathlessness; and overstrain. Each item was dichotomized as ‘no’ vs. ‘yes’. The validity of the scale has been evaluated to be acceptable as reported elsewhere.^25^ Ten-dimensional multivariate trajectory analysis was used to determine the number and shape of trajectories of somatic symptoms across the 5 survey points. We observed the overall trend was that the prevalence of symptoms first decreases and then increases, with age 21 appearing to be a turning point, from which most symptom variables develop in a fairly stable fashion over adulthood. The intercept term was estimated separately for men and women owing to gender differences in the levels of symptoms. A solution with four distinct trajectories resulted in the best fit: ‘constantly low’, ‘increasing’, ‘decreasing’ and ‘constantly high’ somatic symptom load (figure 1).

### Statistical analysis

Multinomial logistic regression analysis was used to examine whether the age 16 variables predicted somatic symptom load trajectory group memberships. First, univariate analyses were run with one predictor variable at the time in the model. Second, multivariate analyses were performed with all predictor variables in the model simultaneously. Forward stepwise selection was used to determine which predictors had the strongest effects. 95% confidence intervals (CIs) were calculated for relative risk ratios. Men and women were examined separately. Analyses were performed using IBM SPSS Statistics 27 software.

## Results

Descriptive statistics of the participants at age 16 are presented in table 1. At follow-up, men compared to women were more often in the low symptom load trajectory (36 vs. 29%), whereas women were more often in the increasing (23 vs. 27%) and decreasing trajectories (23 vs. 27%). A similar proportion of men and women (17%) ended up on a high symptom load trajectory over the follow-up.

**Table 1:** Characteristics of men and women rated at age 16 and trajectories of somatic symptoms over 27 years

	All, *n* = 1001	Men, *n* = 519	Women, *n* = 482
Individual characteristics			
Birth weight			
≥2500 g	932 (94.3)	485 (94.2)	447 (94.5)
<2500 g	56 (5.7)	30 (5.8)	26 (5.5)
Overweight			
No	933 (94.4)	481 (93.9)	452 (95.0)
Yes	55 (5.6)	31 (6.1)	24 (5.0)
Smoking/snuff use			
No	635 (63.9)	336 (65.1)	299 (62.7)
Yes	358 (36.1)	180 (34.9)	178 (37.3)
Ever been drunk			
No	458 (46.1)	253 (48.9)	205 (43.0)
Yes	536 (53.9)	264 (51.1)	272 (57.0)
Early experience of drunkenness at age <14			
No	732 (73.6)	393 (76.0)	339 (70.9)
Yes	263 (26.4)	124 (24.0)	139 (29.1)
Family characteristics			
Contact with mother			
Good	929 (93.5)	491 (95.0)	438 (91.8)
Average/poor	65 (6.5)	26 (5.0)	39 (8.2)
Contact with father			
Good	769 (77.4)	429 (83.0)	340 (71.3)
Average/poor	225 (22.6)	88 (17.0)	137 (28.7)
Parental academic involvement			
Good/average	837 (89.3)	428 (88.6)	409 (90.1)
Poor	100 (10.7)	55 (11.4)	45 (9.9)
Social adversity (composite score)			
No	463 (48.1)	240 (48.0)	223 (48.2)
Yes	500 (51.9)	260 (52.0)	240 (51.8)
Material adversity (composite score)			
No	607 (63.6)	345 (70.4)	262 (56.5)
Yes	347 (36.4)	145 (29.6)	202 (43.5)
School-based factors			
Self-rated school dissatisfaction			
No	814 (81.8)	424 (82.0)	390 (81.6)
Yes	181 (18.2)	93 (18.0)	88 (18.4)
Teacher-rated overall ability			
Good/average	859 (86.3)	426 (82.4)	433 (90.6)
Poor	136 (13.7)	91 (17.6)	45 (9.4)
Teacher-rated peer problems			
No	792 (80.2)	375 (73.2)	417 (87.6)
Yes	196 (19.8)	137 (26.8)	59 (12.4)
Trajectory of somatic symptoms over 26 years			
Constantly low symptom load	328 (32.8)	189 (36.4)	139 (28.8)
Increasing	253 (25.3)	121 (23.3)	132 (27.4)
Decreasing	248 (24.8)	119 (22.9)	129 (26.8)
Constantly high symptom load	172 (17.2)	90 (17.3)	82 (17.0)

*Note*: Figures are numbers (*n*) and percentages (%).

Tables 2 (men) and 3 (women) present the results for individual-, family- and school-based factors as predictors of ‘increasing’ ‘decreasing’ and ‘constantly high’ symptom load compared to ‘constantly low’. In univariate analyses for men, poor contact with father, and teachers’ ratings of poor overall ability and peer problems were significant predictors of ‘increasing’ trajectory membership, whereas smoking and having social adversity predicted the ‘decreasing’ trajectory. Membership in the ‘constantly high’ trajectory was predicted by smoking, alcohol use, poor contact with mother and father, social adversity, dissatisfaction with school and teacher-rated peer problems. In multivariate analyses, social adversity was the significant predictor for both ‘decreasing’ and ‘constantly high’ trajectory groups. ‘Constantly high’ group membership was also predicted by smoking, poor contact with mother and poor school satisfaction.

**Table 2:** Individual, family and school characteristics among boys at age 16 as predictors of trajectories of somatic symptoms over 27 years

Individual, family and school-based factors at age 16	‘Increasing’		‘Decreasing’		‘High’	
	Univariate RRR (95% CI)^a^	Multivariate^b^ RRR (95% CI)^a^	Univariate RRR (95% CI)^a^	Multivariate^b^ RRR (95% CI)^a^	Univariate RRR (95% CI)^a^	Multivariate^b^ RRR (95% CI)^a^
Birth weight ≤2500 g vs. >2500 g	0.64 (0.22–1.86)		1.21 (0.49–2.97)		0.69 (0.22–2.20)	
Current overweight: yes vs. no	1.17 (0.46–2.99)		0.88 (0.32–2.45)		1.18 (0.42–3.31)	
Smoking/snuff use: yes vs. no	1.49 (0.90–2.47)	1.17 (0.67–2.04)	1.75 (1.06–2.88)	1.43 (0.82–2.48)	4.34 (2.53–7.42)	2.72 (1.45–5.12)
Ever been drunk: yes vs. no	0.85 (0.54–1.36)		1.35 (0.85–2.13)		3.24 (1.87–5.62)	
Early experience of drunkenness at age <14 vs. no	0.75 (0.41–1.35)		1.04 (0.60–1.82)		2.78 (1.61–4.79)	
Contact with mother: poor vs. good/average	2.67 (0.63–11.39)	2.68 (0.24–30.29)	2.74 (0.64–11.70)	7.44 (0.84–65.74)	10.61 (2.94–38.27)	10.19 (1.17–88.65)
Contact with father: poor vs. good/average	2.52 (1.27–5.00)		1.79 (0.87–3.70)		6.04 (3.09–11.80)	
Parental academic involvement: poor vs. good/average	1.03 (0.64–1.64)		0.76 (0.47–1.23)		1.58 (0.91–2.74)	
Social adversity (composite score): yes vs. no	1.48 (0.93–2.35)	1.40 (0.85–2.31)	1.85 (1.16–2.96)	1.74 (1.04–2.89)	2.56 (1.49–4.41)	2.75 (1.45–5.22)
Material adversity (composite score): yes vs. no	1.22 (0.73–2.02)		0.89 (0.53–1.51)		1.27 (0.72–2.22)	
Self-reported school dissatisfaction: yes vs. no	1.87 (0.96–3.64)	1.65 (0.78–3.49)	1.19 (0.57–2.47)	0.85 (0.36–2.03)	6.94 (3.69–13.06)	5.22 (2.47–11.04)
Teacher-rated overall ability: poor vs. good/average	1.81 (1.004–3.25)		1.08 (0.57–2.06)		1.52 (0.79–2.94)	
Teacher-rated peer problems: yes vs. no	1.81 (1.07–3.03)		1.37 (0.80–2.35)		1.80 (1.02–3.19)	

*Note*: Relative risk ratios (RRRs) from multinomial logistic regression models with the ‘constantly low symptom load’ trajectory as the reference.

a Reference category ‘Low’.

b Using forward stepwise selection method.

**Table 3:** Individual, family and school characteristics among girls at age 16 as predictors of trajectories of somatic symptoms over 27 years

Individual, family and school-based factors at age 16	‘Increasing’		‘Decreasing’		‘High’	
	Univariate RRR (95% CI)^a^	Multivariate^b^ RRR (95% CI)^a^	Univariate RRR (95% CI)^a^	Multivariate^b^ RRR (95% CI)^a^	Univariate RRR (95% CI)^a^	Multivariate^b^ RRR (95% CI)^a^
Birth weight ≤2500 g vs. >2500 g	0.47 (0.17–1.26)	0.27 (0.08–0.87)	0.24 (0.07–0.86)	0.20 (0.05–0.73)	0.50 (0.16–1.59)	0.19 (0.04–0.92)
Current overweight: yes vs. no	0.38 (0.10–1.45)		0.79 (0.27–2.33)		1.51 (0.53–4.34)	
Smoking/snuff use: yes vs. no	0.97 (0.58–1.61)		1.21 (0.73–2.00)		1.98 (1.13–3.48)	
Ever been drunk: yes vs. no	0.84 (0.52–1.36)		1.14 (0.70–1.86)		1.33 (0.75–2.33)	
Early experience of drunkenness at age <14 vs. no	1.06 (0.62–1.82)		1.22 (0.72–2.07)		1.21 (0.66–2.21)	
Contact with mother: poor vs. good/average	5.00 (1.64–15.30)	5.62 (1.53–20.65)	3.41 (1.07–10.86)	3.13 (0.79–12.40)	2.66 (0.73–9.73)	1.24 (0.23–6.78)
Contact with father: poor vs. good/average	2.16 (1.23–3.78)		1.74 (0.98–3.08)		2.51 (1.35–4.67)	
Parental academic involvement: poor vs. good/average	1.84 (1.12–3.03)	2.05 (1.20–3.50)	2.07 (1.26–3.40)	2.28 (1.32–3.91)	2.37 (1.33–4.23)	2.61 (1.38–5.00)
Social adversity (composite score): yes vs. no	1.77 (1.08–2.88)		1.39 (0.85–2.27)		2.68 (1.49–4.81)	
Material adversity (composite score): yes vs. no	1.29 (0.79–2.10)		0.87 (0.53–1.44)		1.78 (1.01–3.13)	
Self-reported school dissatisfaction: yes vs. no	1.55 (0.76–3.16)	1.32 (0.56–3.11)	1.96 (0.98–3.90)	1.71 (0.74–3.92)	4.07 (2.00–8.25)	4.76 (2.08–10.90)
Teacher-rated overall ability: poor vs. good/average	0.39 (0.15–1.04)		0.76 (0.34–1.72)		1.56 (0.70–3.46)	
Teacher-rated peer problems: yes vs. no	0.59 (0.28–1.26)		1.00 (0.51–1.98)		0.64 (0.27–1.52)	

*Note*: Relative risk ratios (RRRs) from multinomial logistic regression models with the ‘constantly low symptom load’ trajectory as the reference.

a Reference category ‘Low’.

b Using forward stepwise selection method.

For women, poor contact with mother and father, low parental academic involvement and more social adversity were significant predictors of an ‘increasing’ symptom trajectory, whereas poor contact with mother and lower parental academic involvement predicted a ‘decreasing’ trajectory membership in the univariate model. ‘Constantly high’ trajectory membership was predicted by smoking, poor contact with father, low parental academic involvement, social and material adversity and dissatisfaction with school. In multivariate analyses, low parental academic involvement predicted all adverse trajectory groups (‘increasing’, ‘decreasing’ and ‘constantly high’). Furthermore, low birth weight predicted a lower risk for all other trajectory group memberships compared to ‘constantly low’ group. In addition, poor contact with mother predicted ‘increasing’ membership and school dissatisfaction was associated with ‘constantly high’ trajectory membership.

## Discussion

Using a contextual perspective, this 27-year follow-up study examined holistically the association of individual, family and school characteristics among adolescents at age 16, with previously identified trajectories of somatic symptoms until age 43 (‘constantly low’, ‘increasing’, ‘decreasing’ and ‘constantly high’ symptom load).^20^

We found more variability among women, who were more likely to be in either ‘increasing’ or ‘decreasing’ trajectories, whereas men were more likely to have a ‘constantly low’ symptom load trajectory over the follow-up. No differences between men and women were found in the likelihood of being on a ‘constantly high’ trajectory. The higher prevalence of men with a low symptom load trajectory is in accordance with previous research showing that women generally report more somatic symptoms than men.^9^ This difference has been explained by individual and contextual factors such as the higher prevalence of depressive and anxiety disorders among women; gendered life circumstances, e.g. higher exposure to violence and abuse among women than men; the socialization process leading to different readiness to acknowledge and disclose symptoms; as well as to a some extent differences in perception, labelling and description of symptoms.^9^

The settings of school and family on the microlevel were of great importance for the adverse somatic symptom trajectories until midlife, which points to the importance of taking a contextual understanding into account when analysing the development of health complaints. For both men and women, poor contact with mother and dissatisfaction with school predicted adverse symptom trajectories. But there were also differences between genders. Other strong predictors in men were smoking and another aspect of family setting (social adversity), while for women, low birth weight and parents’ low involvement in their school tasks were more salient. Poor contact with mother predicted ‘high’ trajectory in men and ‘increasing’ trajectory in women, although these associations need further evidence from other studies as the CIs were wide due to low number of participants reporting poor contact with mother. Other family predictors were also important, but the relevant indicators differed by gender. For women, parental academic involvement systematically predicted membership in all adverse trajectory groups compared to ‘low’ group, whereas social adversity predicted ‘decreasing’ and ‘high’ trajectory group membership in men. Beyond the single settings of school and family, lack of parental involvement in school constitutes a relation between two settings on the microlevel which is defined as the mesolevel.^12^ Socioecological theory illustrates that seemingly simple associations are extremely context dependent.

A previous study found low intelligence to be associated with a higher predisposition for functional somatic symptoms in adolescents, especially in those adolescents perceiving high parental expectations.^26^ Parental academic involvement likely indicates various phenomena such as parents’ expectations, academic socialization, the social relationship between adolescent and parent as well as cognitive skills, which seem to be important especially to girls’ development. Previous studies using the Northern Swedish Cohort showed that another mesolevel measure, parental interest in their children’s studies, predicted lower risk of entering a ‘moderate stable’ or ‘high decreasing’ trajectory of internalized symptoms, compared with a ‘low stable’ one.^27^ These internalized symptoms often coincide with somatic symptoms^19^ and our previous conclusions regarding them are in line with the present study. The present study shows that it is not just parental academic involvement, but also an individual’s dissatisfaction with school that appears to play an important role in predicting the ‘high’ trajectory group membership later in life.

With respect to the finding that social adversity predicted ‘decreasing’ and ‘high’ trajectory group membership in men, it may be that adverse conditions remain in the ‘high’ group, whereas in the ‘decreasing’ group adversity decreases during the life course. This interpretation requires confirmation in future studies, as well as the suspicion that some of the men experiencing adversity in adolescence underreport somatic symptoms in adult age.

Smoking at age 16 predicted the ‘high’ symptom trajectory in men. Smoking as a risk factor among men was supported in a previous study showing an association between nicotine dependence in adolescence and somatic symptoms in early adulthood.^28^ It is possible that men continued smoking across adulthood while women ceased smoking, for example due to pregnancy,^29^ which further affects the symptom trajectory. In addition, snuff use is more common among men and snuff is very addictive due to the high concentration of nicotine. Our findings regarding social adversity are also consistent with earlier reports showing the association of negative life events^18^ and parenting stress^16^ with the continuance of somatic symptoms among children and adolescents. Finally, gender differences were found in a 5-year follow-up of adolescents, suggesting that smoking predicted somatic symptoms among men, whereas negative life events predicted somatic symptoms among women.^30^ However, in our study, these negative life events (social adversity) were also associated with somatic symptom load for men but not women. There are at least two possible explanations for this gender difference. There might be gender differences in the contribution of later experiences, such as in the incidence of somatic diseases and associated symptom load, on the other hand, our results may be simply caused by chance. These possibilities should be investigated in future studies.

The research showing low birth weight as a risk of adulthood somatic health^31^^,^^32^ gave reason to study birth weight as a predictor of somatic symptoms in this study. Interestingly, we found that for women, low birth weight predicted systematically lower likelihood of all three adverse symptom trajectory group memberships. Low birth weight has been found to decrease the risk of overweight later in life,^33^ and it may be that some of these symptoms are associated with being overweight.^34^^,^^35^ Thus, it may be that the association between low birth weight and somatic symptoms is mediated/moderated by being overweight. However, this issue needs more research as at least a study with different health outcomes was not able to verify the mediating role of obesity in the association between low birthweight and adult health.^32^ In this study, being overweight during adolescence was not a predictor of symptom trajectories, which may indicate that being overweight is a risk factor that manifests more strongly in adults than in adolescents (only 5% of girls in this study were overweight at age 16).

The baseline of this study is at late adolescence, when the entity of ‘internalized symptoms’ of a child has differentiated into adulthood type somatization, depression and anxiety. Still, as shown in an earlier Northern Swedish Cohort study,^19^ these symptoms tend to coincide at baseline as well as during the adult life. The ways and the extent to which the internalized symptoms are mutually associated is interesting, but in the present study, we focus on somatic symptoms and preferred not to include other internalized symptoms as predictors. Also, on basis of the study referred above, we could cautiously conclude that in corresponding analyses with depression and anxiety trajectories as the outcomes, the findings would be largely similar as in the present study with somatic symptoms trajectories.

A major strength of our study is that, it is the first one on this topic with nearly three decades of follow-up. In addition, the attrition rate was very low, and the time span was long enough to reliably assess the development of symptoms from adolescence to adulthood. One limitation of the analysis method is that observations within each trajectory class are assumed to be independent within and between each variable, which may produce a small bias in parameter estimates. Furthermore, as in all observational studies, we cannot rule out the possibility of other unknown or unmeasured confounders. In addition, somatic symptoms reported at midlife may have different origins than those reported during adolescence, for example new-onset somatic diseases. Furthermore, most measures are self-reported and prone to general problems of self-reporting. Teacher assessment of peer relations is limited to school context and is usually only partially in agreement with student assessment.^36^^,^^37^

In conclusion, our findings emphasize the importance of a holistic approach that considers the unique contributions of individual, family and school-based factors in the development of trajectories of somatic symptoms from adolescence to middle age. Such an approach is consistent with the tenets of ecological theories of human development.^12^ The most consistent evidence for symptom trajectories was found for family-related factors. There were gender differences, which need further examinations with larger datasets and more comprehensive analyses.

As to the implications for clinical practice, the present study may help to sharpen the differential diagnostics between general somatic symptoms and symptoms of more specific somatic disorders at different phases of the life course. Moreover, this knowledge may help inform the targeting of preventive measures. Intervention studies are needed to investigate whether early support of families and adolescents would be effective in preventing adverse trajectories of somatic symptoms over the life course.

## Data Availability

The data are not publicly available because the Swedish Data Protection Act (1998:204) does not permit sensitive data on humans (like in our study) to be freely shared. A subset of the dataset may be available after ethical permission and an application to Umeå University. Somatic symptoms among adolescents are common, yet little is known about long-term trajectories of somatic symptoms and the factors in adolescence that shape them. We examined individual, family and school-based factors at age 16 as predictors of trajectories of somatic symptoms over 27 years. The most consistent evidence for symptom trajectories was found for family-related factors. Intervention studies are needed to investigate whether early support of families and adolescents would be effective in preventing adverse trajectories of somatic symptoms over the life course.
